# De novo *SCN1A* missense variant in a patient with Parkinson’s disease

**DOI:** 10.3389/fgene.2024.1496683

**Published:** 2024-11-06

**Authors:** Majed Alluqmani, Abdulfatah M. Alayoubi, Jamil A. Hashmi, Sulman Basit

**Affiliations:** ^1^ Department of Neurology, College of Medicine, Taibah University Medina, Medina, Saudi Arabia; ^2^ Department of Basic Medical Sciences, College of Medicine, Taibah University Medina, Medina, Saudi Arabia; ^3^ Center for Genetics and Inherited Diseases, Taibah University Medina, Medina, Saudi Arabia

**Keywords:** Parkinson’s disease, *SCN1A* gene, mutation, exome sequencing, no epilepsy

## Abstract

**Background:**

Variants in a gene encoding sodium voltage-gated channel alpha subunit 1 (SCN1A) are known to cause a broad clinical spectrum of epilepsy and associated features, including Dravet syndrome (MIM 607208), non-Dravet developmental and epileptic encephalopathy (MIM 619317), familial febrile seizures (MIM 604403), familial hemiplegic migraine (MIM 609634), and generalized epilepsy with febrile seizures (MIM 604403).

**Methods:**

In this study, we examined a patient with Parkinson’s disease (PD) without any clinical manifestations of epilepsy and associated features. Genomic nucleic acid was extracted, and a complete coding sequence of the human genome (whole-exome sequencing) was sequenced. Moreover, Sanger sequencing of variants of interest was performed to validate the exome-discovered variants.

**Results:**

We identified a heterozygous pathogenic missense mutation (c.1498C>T; p.Arg500Trp) in the *SCN1A* gene in the patient using the whole-exome sequencing approach. The onset of PD features in our patient occurred at the age of 30 years. Biochemical investigations were carried out to rule out any secondary cause of the disease, including Wilson's disease or another metabolic disorder. MRI of the brain and spinal images were unremarkable. Moreover, a dramatic response to carbidopa–levodopa treatment was also observed in the patient.

**Conclusion:**

Our results suggest that the pathogenic variant in *SCN1A* may lead to PD features without epilepsy.

## Introduction

Parkinson’s disease (PD) is a neurodegenerative disease characterized by motor and non-motor symptoms. Motor symptoms include bradykinesia, rigidity, and resting tremor, while non-motor symptoms observed in PD are cognitive impairment, constipation, fatigue, sleep disturbance, and depression (Berardelli et al., 2001; Zesiewicz et al., 2003; Aarsland et al., 2009; Baradaran et al., 2013; Williams and Litvan, 2013; Zhu et al., 2016; Loddo et al., 2017; Gironell et al., 2018; Dulski et al., 2019; Balestrino and Schapira, 2020). PD is a complex disease with clinical and genetic heterogeneity. It occurs in autosomal recessive (AR), autosomal dominant (AD), and in X-linked forms. The autosomal recessive forms of Parkinson’s disease can be divided into juvenile [type 2 (MIM 600116) and type 19b (MIM 608375)] and early-onset [type 6 (MIM 605909), type 7 (MIM 606324), type 19b (MIM 615528), type 20 (MIM 615530), and type 23 (MIM 616840)] Parkinson’s disease. Late-onset Parkinson’s disease has been observed mostly in autosomal dominant cases. Genes associated with Parkinson’s disease include *PARK1*, *PARK3*, *PARK7* (*DJ1*), *PARK10*, *PARK12*, *PARK16*, *ATP13A2*, *PINK1*, *DNAJC6*, *GBA*, *HTRA2*, *GIGYF2*, *EI4G1*, *UCHL1*, *SNCA*, *ADH1C*, *PRKN*, *TBP*, *CHCHD2*, *LRRK2*, *ATXN2*, *ATXN8OS*, *VPS13C*, *VPS35*, *MAPT*, *SYNJ1*, *FBXO7*, *PLA2G6*, and *GLUD2* (Hardy, 2010). More than 100 genetic loci have been associated with PD and other forms of Parkinsonism (Dulski et al., 2022). Sequencing of the human genome is becoming cost-effective, which, in turn, leads to more widespread application in research and clinical settings. In particular, whole-exome sequencing (WES) and genome-wide association studies (GWAS) have been instrumental in identifying new variants or combinations of multiple variants (oligo- and polygenic inheritance) in PD patients.

We have applied the WES approach to a patient with a classic PD phenotype. Initially, all known PD-associated genes were screened using the neurodegenerative disease panel for the potential genetic variant. No candidate variant was found in the PD-associated genes. The hypothesis-free unbiased variant prioritization approach identified a pathogenic variant in the *SCN1A* gene. Although variants in the *SCN1A* gene have been reported in various epileptic conditions, our patient was found to be free of any type of epileptic condition.

## Materials and methods

### Blood collection and genomic DNA extraction

**FIGURE 1:**
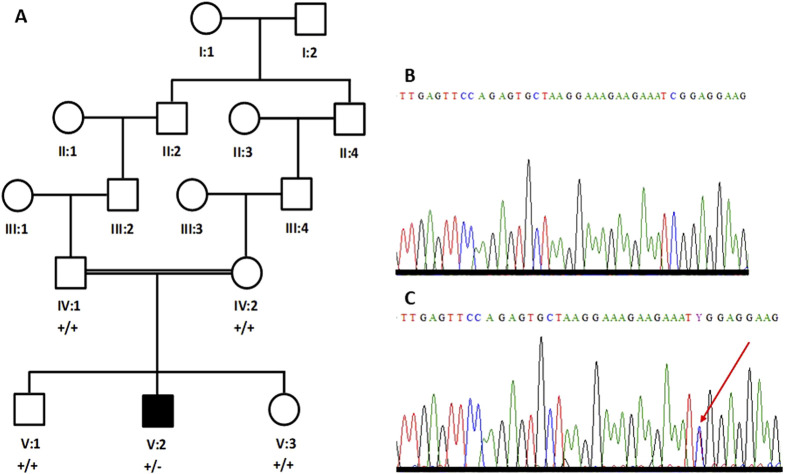
Five-generation pedigree structure of a family with an individual (V:2) with Parkinson’s disease **(A)**. Partial sequence chromatogram of a parent **(B)** and the patient **(C)**. The heterozygous sequence is marked with an arrowhead **(C)**.

Blood samples from five members of one family, including both parents (IV:1 and IV:2), one affected individual (V:2), and two unaffected siblings (V:1 and V:3) were collected in EDTA-containing vacutainers (Figure 1A). Ethical approval for the study was obtained from the research ethics committee (REC) of the College of Medicine, Taibah University Medina, Saudi Arabia. Informed written consent for genetic studies in Arabic and in English was obtained from all participants. Genomic DNA was extracted using the QIAmp DNA isolation kit (Venlo, Netherlands). DNA was quantified using a spectrophotometer.

The patient was examined by a consultant neurologist, and an MRI was performed to detect any structural brain abnormalities.

### Whole-exome sequencing of a DNA sample from the patient

Genomic DNA (250 ng) from the patient (V:2) was used to generate whole-exome amplicons using the Illumina library preparation kit. For this purpose, the Illumina DNA Prep with Exome 2.0 Plus Enrichment kit (San Diego, CA, United Stated) was used. This kit uses bead-linked transposomes and comprehensively covers all the coding exons (37.5-Mb coding content including ≥99% of RefSeq) and variants across public databases (ClinVar and ACMG pathogenic/likely pathogenic variants, COSMIC Cancer Gene Census). DNA libraries were prepared, followed by enrichment and post-enrichment amplification. The whole-exome sequencing protocol used here can be found elsewhere (Rafiullah et al., 2022; Ullah et al., 2022).

Exome libraries were enriched and sequenced on an Illumina NextSeq500 instrument (San Diego, CA, United States). Sequencing reads were collected in base calling (.bcl) files.

### Sanger sequencing of exome-discovered variants of interest

In order to confirm variants detected by exome sequencing, the Applied Biosystems™ (Foster City, CA, United States) capillary electrophoresis (CE)-based genetic analyzer platform and BigDye™ Direct Cycle Sequencing Kit (Foster City, CA, United States) and variant-specific primer sets were used to Sanger sequence-specific amplicons. Unincorporated nucleotides and primers were then removed using the BigDye XTerminator™ Purification Kit. Sequence chromatograms were generated by standard CE. The sequences obtained were read by SeqA software. Moreover, potential candidate variants were also Sanger sequenced in both parents and unaffected individuals to evaluate the segregation of variants.

## Results

### Clinical description

The patient was examined in an adult neurology clinic of King Fahd Hospital Medina by a consultant neurologist. He presented, at the age of 30 years, with stiffness in his left leg while performing his daily activities. He had no prior history of any medical condition or surgical treatment. The asymmetric stiffness worsened with the increasing severity of the pain. Three years later, at the age of 33, intermittent resting tremor of the left hand was observed. Progression of the tremor to the contralateral hand was also observed subsequently. Myerson’s sign (glabellar reflex) was present. Moderate signs of asymmetrical cogwheel rigidity and bradykinesia were also observed. The gait showed typical Parkinson’s disease features (small shuffling steps and reduced arm swing on the left side) and normal postural reflexes. At the age of 36 years, prominent signs of PD such as unintended movements, stiffness, and difficulties with balance and coordination, were observed (Table 1).

**TABLE 1:** Motor and non-motor symptoms identified in the patient with Parkinson’s disease.

Motor symptom	Non-motor symptom
Intermittent resting tremor	Depression
Postural instability	Sleep disorder
Hypophonic speech	Constipation
Cogwheel rigidity	
Bradykinesia	

**FIGURE 2:**
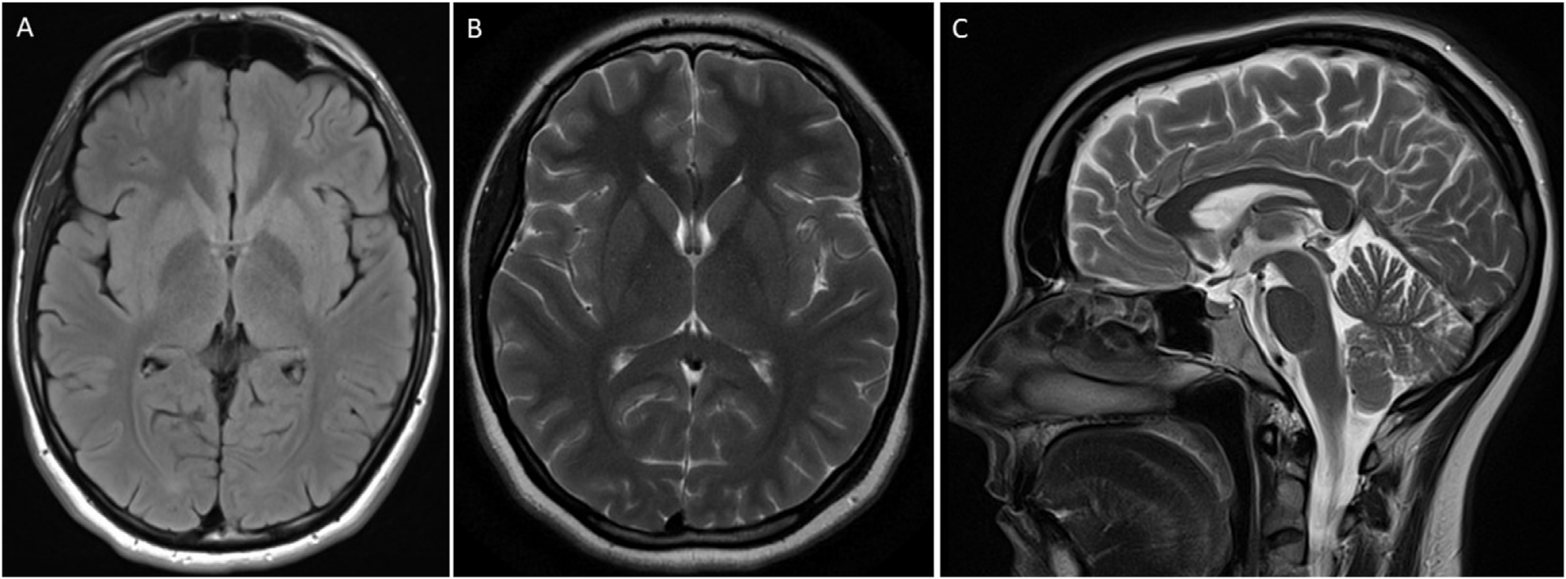
MRI of the brain of an individual showing features of Parkinson’s disease. T2 flair axial **(A)**, brain T2 **(B)**, and brain Sagittal T2 **(C)** were unremarkable.

Neurological examination revealed normal cognition. An oculomotor examination was also unremarkable. Magnetic resonance imaging (MRI) of the brain did not reveal any structural abnormality (Figure 2).

We followed the patient for more than 6 years in an outpatient clinic. He has been on carbidopa–levodopa medication for the last 3 years. Sinemet (25/250 mg) was prescribed for him three times a day; however, no significant improvement was observed.

### WES discovered a missense variant in the *SCN1A* gene

The patient's DNA sample was exome-sequenced to identify the variant(s) underlying the PD phenotype. Analysis of the reads was carried out by aligning the exome reads to the reference genome, followed by variant calling to identify disease-associated variants. The average read length was 150bps, and the average throughput depth of the target regions was 219x. It was observed that 99.8% of reads were mappable. The mean depth of the target regions was 81.6x, and the percentage coverage of the target regions (≥30x) was 92.2%. Variants were filtered using QIAGEN Clinical Insight (QCI) and Genoox Franklin (https://franklin.genoox.com/analysis-tool) tools. The variant interpretation was performed using VarSome, SIFT, PolyPhen, ClinVar, and other prediction tools. Details of the exome data analysis are available elsewhere (Raza et al., 2022; Zaka et al., 2021).

A rare homozygous variant in *NRAP* (NM_006175.5; c.919G>A; p.G307R), compound heterozygous variants in *TTN* (NM_003319.4; c.28328G>A; p.G9443E and NM_003319.4; c.68747A>G; p.Y22916C), and a heterozygous variant in *SCN1A* (NM_001165963.4; c.1498C>T; p.Arg500Trp) genes were filtered and considered to be potential candidate variants for the disease. Variants were classified using ACMG guidelines (Table 2). These variants were further evaluated based on their expression and function using the UCSC genome browser (https://genome.ucsc.edu/) and GeneCards (https://www.genecards.org/). Moreover, PubMed (https://pubmed.ncbi.nlm.nih.gov/) was also searched for the literature related to mutations in these genes. Furthermore, Sanger sequencing was also performed to validate the exome-discovered variants and for segregation analysis within the pedigree. RNA-Seq expression data from GTEx show no expression of *TTN* and *NRAP* in any part of the brain and tibial nerve. Therefore, both *TTN* and *NRAP* variants were not considered candidates for the PD phenotype observed in our patient. *SCN1A* is highly expressed in the different parts of the brain, specifically in the frontal cortex. Moreover, the variant (c.1498C>T) in *SCN1A* is absent in 110 ethnic control DNAs. Therefore, we considered the heterozygous variant in the *SCN1A* gene as a candidate variant for the PD phenotype.

**TABLE 2:** Variants identified and prioritized during whole-exome sequence data analysis.

Gene/variant	gnomAD frequency	CADD	PolyPhen	PyloP	Computed classification
*NRAP*: c.919G>A; p.G307R	0.000004337	Deleterious	Probably damaging	Highly conserved	Uncertain significance
*TTN*: c.28328G>A; p.G9443E	Absent	Deleterious	Probably damaging	Highly conserved	Uncertain significance
*TTN*: c.68747A>G; p.Y22916C	Absent	Deleterious	Probably damaging	Highly conserved	Uncertain significance
*SCN1A*: c.1498C>T; p.Arg500Trp	0.000005577	Deleterious	Probably damaging	Highly conserved	Likely pathogenic

### Sanger sequencing confirmed the *de novo* nature of the *SCN1A* variant

Primers were designed for the missense variant in *SCN1A* (NM_001165963.4; c.1498C>T; p.Arg500Trp) and the variant was bi-directionally sequenced in all the available individuals, including both parents. The patient’s DNA carries the variant in the heterozygous state (Figure 1C); however, the variant is not present in either parent’s DNA (Figure 1B). Therefore, it is considered a *de novo* variant.

## Discussion

PD is the second most common neurodegenerative disease and is characterized by misfolding and aggregation of α-synuclein in the cytoplasm of the neurons (Lewy bodies), leading to progressive loss of dopaminergic (DA) neurons in the pars compacta of the substantia nigra (SNpc) (Balestrino and Schapira, 2020). DA neurons are the main source of dopamine in the mammalian CNS (Chinta and Andersen, 2005). Therefore, they are essential for voluntary movement, cognition, emotion, working memory, and reward pathways (Chinta and Andersen, 2005; Salgado-Pineda et al., 2005; Bromberg-Martin et al., 2010; McNamara et al., 2014). Insufficient dopamine levels not only cause disabling motor symptoms but also lead to mood changes and memory loss (Chinta and Andersen, 2005). DA neurons have spontaneous action potential firing (pacemaker properties) ability (Puopolo et al., 2007; Guzman et al., 2009). This distinct electrical activity is important for dopamine secretion. Ion channel activities, including voltage-gated sodium channels, control the pacemaker frequency (Tucker et al., 2012; Ilin et al., 2021). Therefore, we hypothesize that mutations affecting the function or expression of voltage-gated sodium channels may be responsible for neurological disorders, including PD.

PD segregates in both autosomal dominant and autosomal recessive forms. Mutations in several genes have been found as the underlying cause of PD (Klein and Westenberger, 2012). Genetic mutations underlying PD or PD risk factors can be delineated using gene mapping, candidate gene sequencing, genome-wide association studies, or whole-exome and whole-genome sequencing approaches. Gene mapping, exome, and genome sequencing approaches usually use hypothesis-free approaches to identify the genetic variant underlying the phenotype.

Here, in this study, we also used a hypothesis-free approach and sequenced the complete coding part of the human genome in a DNA sample from a patient with a classical PD phenotype. We identified a deleterious heterozygous missense variant (c.1498C>T; p.Arg500Trp) in a *SCN1A* gene in a patient with a typical PD phenotype. Mutations in the *SCN1A* gene are known to cause autosomal dominant Dravet syndrome (MIM 607208), non-Dravet developmental and epileptic encephalopathy (MIM 619317), familial febrile seizures (MIM 604403), familial hemiplegic migraine (MIM 609634), and generalized epilepsy with febrile seizures (MIM 604403). Seizures and ophthalmological abnormalities (nystagmus—visual impairment) are hallmarks of the *SCN1A* phenotype. For instance, the same heterozygous variant (c.1498C>T; p.Arg500Trp) in the *SCN1A* gene has been reported in a patient with refractory seizures and cognitive impairment (Nashabat et al., 2019). However, our patient is free of seizures and eye abnormalities. Moreover, the brain MRI of the patient did not show any structural abnormality. The same disease-causing variant underlying different clinical phenotypes may be due to variable penetrance and expressivity. Studies have shown that modifier genes, genetic background, and complex genetic and environmental interactions can cause phenotypic variation (Kammenga, 2017).

Therefore, we hypothesize that *de novo* variants of *SCN1A* are the underlying cause of autosomal dominant PD. However, there are some limitations, and further research is needed to validate and confirm our findings. For instance, WES analysis is limited to variants in the coding part and flanking regions of exons. Homozygous or compound heterozygous variants in the non-coding part and copy number variations are missed by WES analyses.

## Data Availability

The original contributions presented in the study are publicly available. This data can be found here: https://www.ncbi.nlm.nih.gov/biosample/44076318. The accession number is SAMN44076318.

## References

[B1] Aarsland D. Marsh L. Schrag A. (2009). Neuropsychiatric symptoms in Parkinson’s disease. Mov. Disord. 24 (15), 2175–2186. 10.1002/mds.22589 19768724 PMC2787875

[B2] Balestrino R. Schapira A. H. V. (2020). Parkinson disease. Eur. J. Neurol. 27, 27–42. 10.1111/ene.14108 31631455

[B3] Baradaran N. Tan S. N. Liu A. Ashoori A. Palmer S. J. Wang Z. J. (2013). Parkinson’s disease rigidity: relation to brain connectivity and motor performance. Front. Neurol. 4, 67. 10.3389/fneur.2013.00067 23761780 PMC3672800

[B4] Berardelli A. Rothwell J. C. Thompson P. D. Hallett M. (2001). Pathophysiology of bradykinesia in Parkinson’s disease. Brain 124 (11), 2131–2146. 10.1093/brain/124.11.2131 11673316

[B5] Bromberg-Martin E. S. Matsumoto M. Hikosaka O. (2010). Dopamine in motivational control: rewarding, aversive, and alerting. Neuron 68 (5), 815–834. 10.1016/j.neuron.2010.11.022 21144997 PMC3032992

[B6] Chinta S. J. Andersen J. K. (2005). Dopaminergic neurons. Int. J. Biochem. Cell Biol. 37 (5), 942–946. 10.1016/j.biocel.2004.09.009 15743669

[B7] Dulski J. Schinwelski M. Konkel A. Grabowski K. Libionka W. Wa̧ż P. (2019). The impact of subthalamic deep brain stimulation on sleep and other non-motor symptoms in Parkinson’s disease. Park. Relat. Disord. 64, 138–144. 10.1016/j.parkreldis.2019.04.001 30975618

[B8] Dulski J. Uitti R. J. Ross O. A. Wszolek Z. K. (2022). Genetic architecture of Parkinson's disease subtypes - review of the literature. Front. Aging Neurosci. 14, 1023574. PMID: 36337703; PMCID: PMC9632166. 10.3389/fnagi.2022.1023574 36337703 PMC9632166

[B9] Gironell A. Pascual-Sedano B. Aracil I. Marín-Lahoz J. Pagonabarraga J. Kulisevsky J. (2018). Tremor types in Parkinson disease: a descriptive study using a new classification. Parkinson’s Dis. 2018, 4327597. 10.1155/2018/4327597 30363956 PMC6186312

[B10] Guzman J. N. Sánchez-Padilla J. Chan C. S. Surmeier D. J. (2009). Robust pacemaking in substantia nigra dopaminergic neurons. J. Neurosci. 29 (35), 11011–11019. 10.1523/JNEUROSCI.2519-09.2009 19726659 PMC2784968

[B11] Hardy J. (2010). Genetic analysis of pathways to Parkinson disease. Neuron 68 (2), 201–206. PMID: 20955928; PMCID: PMC2997424. 10.1016/j.neuron.2010.10.014 20955928 PMC2997424

[B12] Ilin V. A. Bai Q. Watson A. M. Volgushev M. Burton E. A. (2021). Mechanism of pacemaker activity in zebrafish DC2/4 dopaminergic neurons. J. Neurosci. 41 (18), 4141–4157. 10.1523/JNEUROSCI.2124-20.2021 33731451 PMC8176752

[B13] Kammenga J. E. (2017). The background puzzle: how identical mutations in the same gene lead to different disease symptoms. FEBS J. 284 (20), 3362–3373. 10.1111/febs.14080 28390082

[B14] Klein C. Westenberger A. (2012). Genetics of Parkinson's disease. Cold Spring Harb. Perspect. Med. 2 (1), a008888. PMID: 22315721; PMCID: PMC3253033. 10.1101/cshperspect.a008888 22315721 PMC3253033

[B15] Loddo G. Calandra-Buonaura G. Sambati L. Giannini G. Cecere A. Cortelli P. (2017). The treatment of sleep disorders in Parkinson’s disease: from research to clinical practice. Front. Neurol. 8, 42. 10.3389/fneur.2017.00042 28261151 PMC5311042

[B16] McNamara C. G. Tejero-Cantero Á. Trouche S. Campo-Urriza N. Dupret D. (2014). Dopaminergic neurons promote hippocampal reactivation and spatial memory persistence. Nat. Neurosci. 17 (12), 1658–1660. 10.1038/nn.3843 25326690 PMC4241115

[B17] Nashabat M. Al Qahtani X. S. Almakdob S. Altwaijri W. Ba-Armah D. M. Hundallah K. (2019). The landscape of early infantile epileptic encephalopathy in a consanguineous population. Seizure 69, 154–172. Epub 2019 Apr 27. PMID: 31054490. 10.1016/j.seizure.2019.04.018 31054490

[B18] Puopolo M. Raviola E. Bean B. P. (2007). Roles of subthreshold calcium current and sodium current in spontaneous firing of mouse midbrain dopamine neurons. J. Neurosci. 27 (3), 645–656. 10.1523/JNEUROSCI.4341-06.2007 17234596 PMC6672803

[B19] Rafiullah R. Albalawi A. M. Alaradi S. R. Alluqmani M. Mushtaq M. Wali A. (2022). An expansion of phenotype: novel homozygous variant in the MED17 identified in patients with progressive microcephaly and global developmental delay. J. Neurogenet. 12, 108–114. Epub ahead of print. PMID: 36508181. 10.1080/01677063.2022.2149748 36508181

[B20] Raza R. Ullah A. Haider N. Krishin J. Shah M. Khan F. U. (2022). Exome sequencing reveals the first intragenic deletion in ABCA5 underlying autosomal recessive hypertrichosis. Clin. Exp. Dermatol 12, 1137–1143. Epub ahead of print. PMID: 35150007. 10.1111/ced.15128 35150007

[B21] Salgado-Pineda P. Delaveau P. Blin O. Nieoullon A. (2005). Dopaminergic contribution to the regulation of emotional perception. Clin. Neuropharmacol. 28 (5), 228–237. 10.1097/01.wnf.0000185824.57690.f0 16239763

[B22] Tucker K. R. Huertas M. A. Horn J. P. Canavier C. C. Levitan E. S. (2012). Pacemaker rate and depolarization block in nigral dopamine neurons: a somatic sodium channel balancing act. J. Neurosci. 32 (42), 14519–14531. 10.1523/JNEUROSCI.1251-12.2012 23077037 PMC3494994

[B23] Ullah A. Shah A. A. Alluqmani M. Haider N. Aman H. Alfadhli F. (2022). Clinical and genetic characterization of patients segregating variants in KPTN, MINPP1, NGLY1, AP4B1, and SON underlying neurodevelopmental disorders: genetic and phenotypic expansion. Int. J. Dev. Neurosci. 82 (8), 788–804. Epub 2022 Oct 11. PMID: 36181241. 10.1002/jdn.10231 36181241

[B24] Williams D. R. Litvan I. (2013). Parkinsonian syndromes. Continuum 19 (5), 1189–1212. 10.1212/01.CON.0000436152.24038.e0 24092286 PMC4234134

[B25] Zaka A. Shahzad S. Rao H. Z. Kanwal S. Gul A. Basit S. (2021). An intrafamilial phenotypic variability in Ellis-Van Creveld syndrome due to a novel 27 bps deletion mutation. Am. J. Med. Genet. A 185 (10), 2888–2894. Epub 2021 May 26. PMID: 34037314. 10.1002/ajmg.a.62360 34037314

[B26] Zesiewicz T. A. Baker M. J. Wahba M. Hauser R. A. (2003). Autonomic nervous system dysfunction in Parkinson’s disease. Curr. Treat. Options Neurol. 5 (2), 149–160. 10.1007/s11940-003-0005-0 12628063

[B27] Zhu M. Li M. Ye D. Jiang W. Lei T. Shu K. (2016). Sensory symptoms in Parkinson’s disease: clinical features, pathophysiology, and treatment. J. Neurosci. Res. 94 (8), 685–692. 10.1002/jnr.23729 26948282

